# Community health workers during the Ebola outbreak in Guinea, Liberia, and Sierra Leone

**DOI:** 10.7189/jogh-08-020601

**Published:** 2018-12

**Authors:** Nathan P Miller, Penelope Milsom, Ginger Johnson, Juliet Bedford, Aline Simen Kapeu, Abdoulaye Oumar Diallo, Kebir Hassen, Nuzhat Rafique, Kamrul Islam, Robert Camara, Joseph Kandeh, Chea Sanford Wesseh, Kumanan Rasanathan, Jerome Pfaffmann Zambruni, Heather Papowitz

**Affiliations:** 1 UNICEF, New York, New York, USA; 2 Anthrologica, Oxford, United Kingdom; 3 UNICEF, Conakry, Guinea; 4 UNICEF, Freetown, Sierra Leone; 5 UNICEF, Monrovia, Liberia; 6 Republic of Guinea Ministry of Health, Conakry, Guinea; 7 Republic of Sierra Leone Ministry of Health and Sanitation, Freetown, Sierra Leone; 8 Republic of Liberia Ministry of Health, Monrovia, Liberia

## Abstract

**Background:**

The role of community health workers (CHWs) in the West Africa Ebola outbreak has been highlighted to advocate for increasing numbers of CHWs globally to build resilience, strengthen health systems, and provide emergency response capacity. However, the roles CHWs played, the challenges they faced, and their effectiveness during the outbreak are not well documented. This study assessed the impact of Ebola on community-based maternal, newborn, and child health (MNCH) services, documented the contribution of CHWs and other community-based actors to the Ebola response, and identified lessons learned to strengthen resilience in future emergencies.

**Methods:**

This mixed methods study was conducted in Guinea, Liberia, and Sierra Leone, with data collected in four Ebola-affected districts of each country. Qualitative data were collected through in-depth interviews and focus group discussions with stakeholders at national, district, and community levels. Quantitative program data were used to assess trends in delivery of community-based MNCH services.

**Results:**

There was a sharp decline in MNCH service provision due to weak service delivery, confusion over policy, and the overwhelming nature of the outbreak. However, many CHWs remained active in their communities and were willing to continue providing services. When CHWs received clear directives and were supported, service provision rebounded. Although CHWs faced mistrust and hostility from community members because of their linkages to health facilities, the relationship between CHWs and communities proved resilient over time, and CHWs were more effectively able to carry out Ebola-related activities than outsiders. Traditional birth attendants, community health committees, community leaders, and traditional healers also played important roles, despite a lack of formal engagement or support. Service delivery weaknesses, especially related to supply chain and supervision, limited the effectiveness of community health services before, during, and after the outbreak.

**Conclusions:**

CHWs and other community-level actors played important roles during the Ebola outbreak. However, maintenance of primary care services and the Ebola response were hampered because community actors were engaged late in the response and did not receive sufficient support. In the future, communities should be placed at the forefront of emergency preparedness and response plans and they must be adequately supported to strengthen service delivery.

The epidemic of Ebola Virus Disease (EVD) that began in Guinea in December 2013 spread across West Africa affecting thousands of people in Guinea, Liberia, and Sierra Leone. The cumulative number of confirmed, probable, and suspected EVD cases in the three most-affected countries was 28 616 and the number of confirmed, probable, and suspected deaths was 11 310 [1]. The Ebola crisis highlighted and exacerbated persistent weaknesses and vulnerabilities in the national health systems, which did not have the capacity to respond to an outbreak of that scale or to maintain the provision of essential life-saving health services [2-6].

Following the outbreak, the work of community health workers (CHWs - some terms have been standardized in this paper for simplicity and readability. We use the terms community health worker (CHW), traditional birth attendant (TBA), and community health committee (CHC) even though these are not the terms universally used across all three countries. Likewise, we refer to the Ministry of Health (MoH) of each country, although the official name in Sierra Leone is the Ministry of Health and Sanitation.) in the West Africa Ebola outbreak has been highlighted to advocate for increasing the numbers of CHWs globally to build resilience (We use the definition of a resilient health system suggested by Kruk and colleagues: “*The capacity of health actors, institutions, and populations to prepare for and effectively respond to crises; maintain core functions when a crisis hits; and, informed by lessons learned during the crisis, reorganise if conditions require it*” [7]), strengthen health systems, and provide capacity to respond to future emergencies [8-10]. However, there remain significant gaps in understanding regarding the roles CHWs played, the challenges they faced, and their effectiveness during the outbreak. This study documented the work of CHWs and other community-level actors during the Ebola outbreak to inform efforts to strengthen community health systems and build resilience. This article provides a summary of results for the three countries. More detailed country-specific findings are available in a series of United Nations Children’s Fund (UNICEF) working papers [11-13].

## METHODS

We conducted a mixed methods study using in-depth interviews (IDIs), focus group discussions (FGDs), and analysis of routine program data to understand the experience of CHWs and other community-based actors during the Ebola outbreak in Guinea, Liberia, and Sierra Leone.

### Objectives

The study had four objectives:

- To assess the effect of EVD on community-based maternal, newborn, and child health (MNCH) service delivery and care seeking.
- To document and assess the contribution of CHWs and other community-based actors to the EVD response.
- To identify how CHWs and other community-based actors could have been more effectively used and supported during the outbreak.
- To determine lessons learned and recommendations for early recovery, health systems strengthening, and improving resilience.

### Site selection

Selection of research sites for the study was based on three key factors:

- High EVD transmission;
- Established integrated community case management of childhood illness (iCCM) or other community-level MNCH programming by CHWs prior to the Ebola outbreak; and
- Distance from a primary healthcare facility (with representation of communities that were closer and further from health facilities).

Sites were purposively selected to ensure variability in urban, peri-urban, and rural locations and for diversity in population groups (eg, ethnicity, religion).

**Figure 1:**
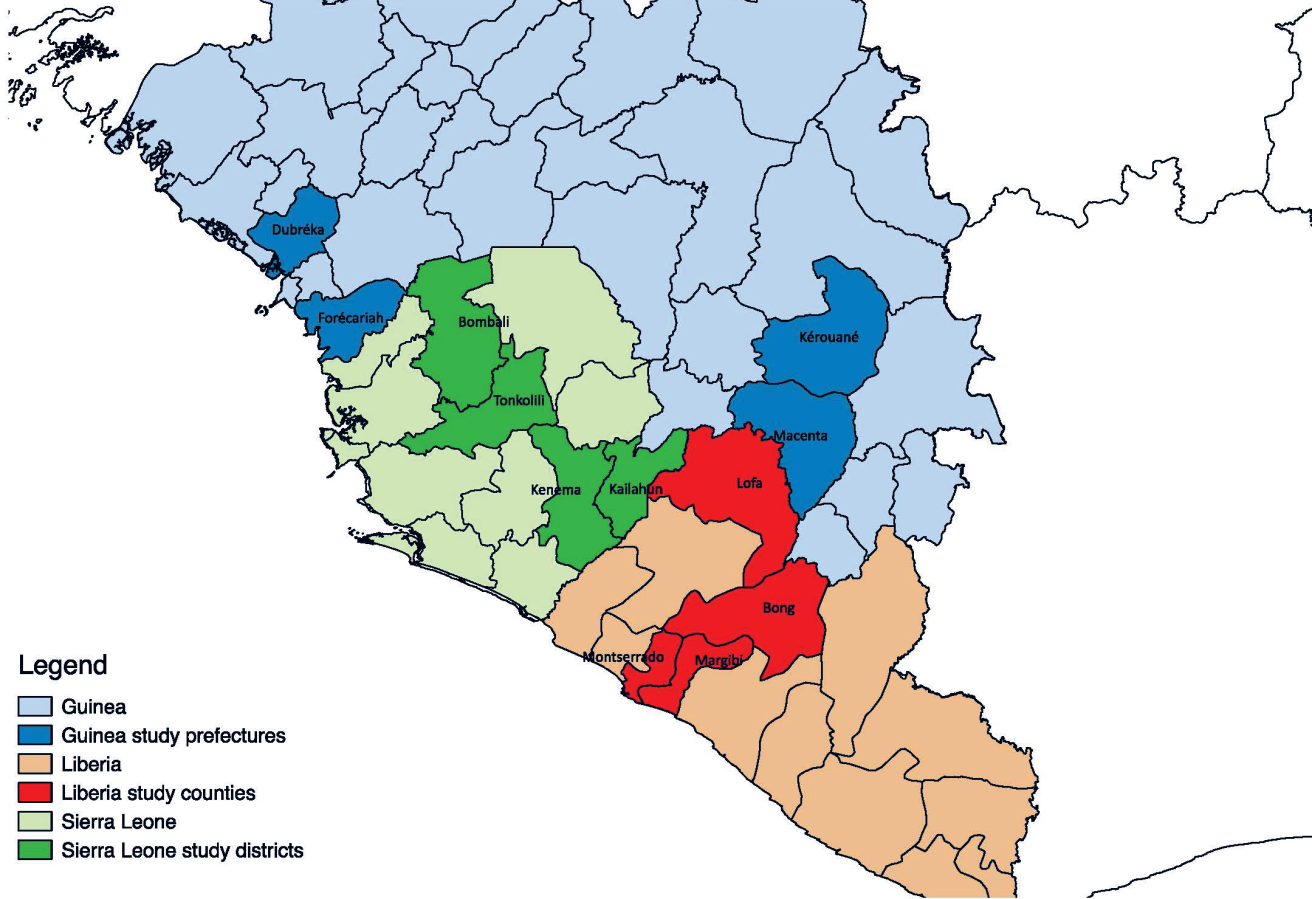
Map of the study districts.

In consultation with the Ministries of Health (MoH) of each country and in line with the above criteria, four districts were selected in Guinea (Dubréka, Forécariah, Macenta and Kérouané), Liberia (Lofa, Montserrado, Margibi, and Bong), and Sierra Leone (Kenema, Kailahun, Bombali and Tonkolili). Within each district, two communities were selected for qualitative data collection. **Figure 1** shows the study districts.

### Qualitative data

#### Participants and recruitment

Study participants were selected using purposive, non-probability sampling. Respondents were selected if they had detailed knowledge of community MNCH services (eg, They were involved in community MNCH policy, implementation, or supervision, or were recipients of CHW services) before, during, or after Ebola, and/or with the community-level Ebola response. Respondents included representatives from the MoH, United Nations agencies, international non-governmental organizations (INGOs), national NGOs, and traditional healers (THs) unions. At the district level, respondents included key implementing partners, UNICEF staff, district health personnel, local government authorities, and TH associations. At the community-level, informants included community leaders, caregivers of children under five, CHWs, traditional birth attendants (TBAs), officers in charge of health facilities, maternal and child health aides in health facilities, members of community health committees (CHCs), and EVD survivors.

#### Data collection

Data collection was conducted between February and August 2016 (February-March in Liberia; May-June in Sierra Leone; and July-August in Guinea). Data were collected through IDIs and FGDs. Field researchers used semi-structured IDI and FGD guides that were broadly based on four study objectives given above. Within each of these broad themes, questions and probes were informed by the iCCM Benchmarks Framework [14], which ensured that key service delivery components (coordination and policy setting, costing and financing, human resources, supply chain management, service delivery and referral, communication and social mobilization, supervision and performance quality assurance, monitoring and evaluation and health information systems) would be captured in the data. Separate guides were used for each type of respondent, but key themes were addressed in each interview and FGD, which allowed analysis of themes across participant groups and fieldsites. IDI and FGD participants were identified by UNICEF, MoH, and district health officials or by community leaders based on their experiences with the Ebola outbreak and community- and facility-based health services. The research was designed to facilitate input from multiple stakeholders using a phased approach, so that issues raised by one group of interlocutors could be discussed with other groups of stakeholders as appropriate. This ensured the collation of in-depth material and the rigour of its validation and triangulation. Research teams were expected to complete approximately 44 interviews and 16 FGDs (with 6-8 participants each) per country.

Data collection was carried out by one lead researcher from Anthrologica (a research organisation specializing in applied anthropology in global health), and one local research assistant in each country. The lead researcher led the interviews and FGDs, with the assistant providing translation when necessary and taking notes. Research assistants were experienced qualitative researchers who had previously worked with Anthrologica, and were trained prior to data collection on the study objectives and procedures.

#### Data analysis

At the end of data collection, audio recordings of all interviews and FGDs were transcribed and translated into English as necessary. The transcripts were reviewed for accuracy and were cross-referenced with the research team’s field notes. Any areas of inconsistency were resolved after additional review of the original audio file.

Dominant themes were identified through the systematic review of interview and FGD transcripts. Thematic analysis was primarily deductive, based on the research questions and service delivery components (eg, policy, coordination, training, supply chain, supervision, social mobilization). Additional themes were identified inductively from the data. All qualitative data were coded and analyzed by hand. Two researchers coded an initial subset of the data. The two sets of codes were then compared and any discrepancies were discussed and consensus was reached on the codes that were used on the rest of the data.

### Quantitative data

Routine quantitative data on community-based MNCH activities from January 2013 to December 2015 were requested from the MoH or the NGO implementing partner for each of the study districts. Data were collected by the MoH and NGO partners in each district according to their own data collection procedures. Because this study was retrospective, we were limited to using pre-existing quantitative data, and no special data collection procedures were put in place for the study.

The highest priority indicator that was requested was the number of children treated for iCCM-related illnesses. This indicator was prioritized because of the relatively high level of health system support required to implement iCCM (eg, continuous supply of commodities and regular supervision). The other indicators collected varied by district depending on what services were provided by CHWs. Indicators were included if the data were available in the database for the period of interest and were an indicator of MNCH services delivered to community members (ie, related to patient contacts). Final included indicators were determined during data collection and are shown in the results.

The data obtained were reviewed and triangulated with qualitative data on service delivery from the same districts. Data that did not pass a check for completeness and consistency were discarded. The indicators were presented as a monthly trend, which allowed for an assessment of the levels of service delivery before, during, and after the Ebola outbreak.

### Ethical considerations

At the start of each interview and FGD, it was made clear to all potential participants that their involvement was optional and voluntary, and would not affect any future medical services and/or benefits received. All research participants gave informed consent by signature or verbal agreement and thumbprint. Ethical approval was obtained from the institutional review boards of the MoH of Guinea, Liberia, and Sierra Leone.

## RESULTS

At the end of data collection 124 IDIs and 78 FGDs were conducted, with a total of 582 participants (193 in Guinea, 205 in Liberia, and 184 in Sierra Leone). The number and distribution of participants by respondent type, country, and data collection method are presented in **Table 1**.

**Table 1:** Number of study participants by type of respondent, country, and data collection method

	Guinea			Liberia			Sierra Leone		
**Type of respondent**	**IDIs**	**FGDs**	**Total participants**	**IDIs**	**FGDs**	**Total participants**	**IDIs**	**FGDs**	**Total participants**
National-level stakeholders*	13†	–	15	18	–	28	13†	–	14
District-level stakeholders‡	16†	–	21	16	–	24	19	1	25
Health facility workers	6†	–	7	–	–	–	8†	–	11
CHWs§	-	8	38	–	8	29	3	5	35
TBAs	4^b^	–	5	–	5	18	1	1	5
Community leaders||	-	8	48	–	8	51	–	8	42
Caregivers of children under 5 years	-	8	49	–	8	55	–	8	40
Ebola survivors¶	4†	–	10	–	–	–	2	2	12
Total	43	24	193	34	29	205	46	25	184

IDI – in-depth interviews, FGD – focus-group discussions, CHW – community health worker, TBA – traditional birth attendant

*Ministry of Health, United Nations, international non-governmental organizaitons, traditional healer unions.

†Some IDIs were conducted with more than one person, which is why the number of participants is larger than the number of IDIs.

‡District health teams, district government, UNICEF, INGOs, national NGOs, and TH associations.

§In Liberia, some CHC members were also included in CHW FGDs. In Guinea and Sierra Leone, CHC members were included in community leader FGDs.

||Traditional leaders, religious leaders, women leaders, youth leaders, traditional healers, and CHC members.

¶Additional Ebola survivors also participated in FGDs with other participant groups (eg, TBAs).

Quantitative program data was obtained for all four districts in Sierra Leone, but only for one district in Liberia and none in Guinea. Data for the other three districts in Liberia did not pass the assessment of quality and consistency, so were not used. In Guinea, we were unable to attain monthly data on community-based health services.

### Community-based MNCH services pre-Ebola

#### Service delivery

Prior to Ebola, the governments of Guinea, Liberia, and Sierra Leone recognized the importance of community-based MNCH services to improve access to essential care. National policies were launched in 2011 in Liberia and in 2012 in Guinea and Sierra Leone that defined the roles, responsibilities, selection criteria, training, supervision, and reporting for CHWs. The policies were intended as a framework for harmonizing programming under MoH authority and oversight. However, because of a reliance on donor funding and INGO implementing partners, in reality there was a complex landscape of stand-alone projects and limited MoH oversight.

CHWs in the three countries worked as volunteers, but received small travel allowances and some non-financial incentives, such as boots, rain gear, and flashlights. Although CHWs worked in their home communities, they usually covered several additional communities, often long distances from their home.

The services provided by CHWs varied by country, district, and implementing partner. The most common services delivered included iCCM; community sensitization and health promotion; screening for childhood undernutrition; childhood immunization; distribution and promotion of insecticide-treated bednets; and referral for danger signs in children and pregnant or postpartum women. However, most CHWs were not implementing the full package of services. In Guinea for example, CHWs were providing community case management (CCM) of malaria, but were not treating pneumonia or diarrhea.

A number of challenges were highlighted that hindered effective implementation of community health services. These included:

- Difficulty recruiting CHWs satisfying the minimum selection criteria;
- Inadequate numbers of CHWs in hard-to-reach communities;
- Lack of transparency in the CHW selection process;
- Weak links between CHWs and the formal health system;
- Frequent stock-outs of commodities and supplies;
- Cultural beliefs and practices inhibiting the utilization of MNCH services and lack of knowledge of available services;
- Lack of integration of community data into the health management information system; and
- Weak supervision, monitoring, and evaluation of CHW activities.

Supply chain management and supportive supervision were frequently highlighted as key challenges in all three countries. Common supply chain bottlenecks included shortages of drugs at the national level, lengthy bureaucratic processes to procure drugs from national warehouses, and health facilities choosing to keep medication at the facility rather than supply CHWs. As a result of persistent stock-outs, iCCM had effectively ceased in three of the four study districts in Liberia prior to the Ebola outbreak. In Guinea and Sierra Leone the situation was less severe, with generally functioning iCCM/CCM programs that faced occasional drug stock-outs. Supervision proved challenging because of shortages of health staff and the fact that many CHWs were required to travel long distances to attend supervision meetings at the health facilities and they often incurred travel costs that exceeded their monthly incentive.

CHWs frequently reported that they were not adequately incentivized. Besides the lack of financial incentive, many CHWs had not been supplied with basic equipment such as rain gear and ID cards. A CHW in Guinea explained the challenges that CHWs faced in carrying out their work:

“*We have four challenges that we face in implementing services in the communities. The first challenge is the lack of motivation fees. The second is the lack of mobility. They gave us bicycles, but the places where we are to do the activities are in the hills...Some districts are 12 miles distant. The third challenge is the lack of protection materials...We need rain boots and rain coats but up to now we have not got them. The fourth challenge is the non-repetitive training. If they give us one training it will take one year before having another.*”

CHWs also faced challenges in balancing their non-CHW paid work with voluntary CHW activities. Some CHWs admitted that they were not always available for community health duties because of other commitments. Both health workers and NGO staff suggested that they were not able to hold CHWs to account for their community health responsibilities because they were not being paid. National- and prefecture-level stakeholders remained concerned that CHW interventions were unsustainable without some form of increased incentive.

#### Care seeking and referral compliance

In many communities, caregivers appeared well informed about CHW services and reported that they would first seek care from a CHW if their child was ill. Conversely, in communities where CHWs did not have a routine supply of medication, CHWs were considered to primarily provide first aid before referring the child to a health facility for treatment. In communities that did not have a resident CHW, caregivers had less knowledge about and experience of their services.

Many barriers to referral compliance were reported. These included distance from home to the health facility, poor road conditions, limited transport, and the cost of transport. A perception of poor quality care provided at the health facility also caused community members to be reluctant to seek care outside the community. Frequently caregivers did not follow referral advice but sought care from alternative community-based providers, such as THs. Stakeholders described several community-based strategies that had been adopted to facilitate referral, including the CHW accompanying the caregiver and child to the facility, and the community pooling funds to pay for transport, but the impact and scale of such initiatives remained unclear.

#### Other community-based actors

Although TBAs traditionally carried out deliveries in their communities, each country had enacted policies to promote facility-based delivery. TBAs were prohibited from assisting deliveries in the community, and were instead enlisted as “maternal health promoters” to encourage women to seek family planning, antenatal care, and plan for facility-based deliveries. However, despite these policies, community members and TBAs acknowledged that routine deliveries had continued to be performed in the community, especially in communities that were further from a health facility. Although TBAs were recognized as an official health cadre in all three countries, they received limited support, not only in terms of incentive, but also with regards to training, supervision, and material resources. TBAs and CHWs frequently reported that they worked “hand-in-hand” and perceived their roles as being distinct but complementary, and it was broadly understood that CHWs had a supervisory role over TBAs.

Communities, particularly in rural areas, strongly believed in “traditional” practices and THs were often the first person caregivers would seek treatment from for ill children. THs were largely excluded from government policy, were not engaged by the health system, and did not formally interact with CHWs. Many expressed frustration at what they considered to be neglect by the government.

Each country had some version of CHCs that were recognized in MoH policies, but whose roles were not clearly defined and whose level of activity in communities varied. In communities with CHCs, they were made up of community leaders, religious leaders, women’s leaders, and youth leaders. Their activities included health promotion, community mobilization, selection and supervising of CHWs and TBAs, liaising with health facilities, promoting facility-based deliveries, promoting the use of CHWs, accompanying ill people to facilities, and pooling community funds to finance emergency transport and treatment. Where CHCs were active, they reportedly worked closely with the CHWs, supporting their activities and amplifying their health promotion messages. CHCs tended to be more active if they were supported by an NGO for a specific project and stakeholders expressed concern that CHCs were only active if financially motivated.

Community leaders played an important function brokering relations between the community and CHWs, and CHWs strongly emphasized the need for community leaders to advocate for them. As one CHW in Sierra Leone affirmed, “*The chief is going to be your ambassador and has to introduce you to the community*.” Community leaders also enforced the community bylaws, including the prohibition of home deliveries.

### Continuity of MNCH services during Ebola

#### Service delivery

The extent to which community-based MNCH services continued during Ebola varied by country, district, and phase of the outbreak. The majority of stakeholders engaged in this study concluded that CHWs were willing to continue their regular activities during the Ebola outbreak, motivated by a commitment to their communities and a desire to contribute to “*a cause larger than themselves*.” However, the ability of CHWs to continue providing services was largely dependent on the level of support they received. In all three countries, case management services largely stopped or drastically declined in the early months of the outbreak. Reasons for withdrawal or reduction of iCCM/CCM services included CHWs’ fear of becoming infected, reorientation towards Ebola-related activities, lack of support, and/or because of a clear directive from an implementing partner or health facility to cease providing treatment.

In recognition of the need to restart community-based services and the fact that CHWs were at high-risk of exposure to Ebola, the WHO and UNICEF, in collaboration with the MoH in each country, developed a “no touch policy” that recommended that CHWs avoid all physical contact with patients and base their assessments solely on patient history and observations [15]. The “no touch policy” was adopted in all three countries and training of CHWs on the protocol began in late 2014. Many CHWs experienced a long period between when the outbreak was declared in their district to when they received training. In Macenta, Guinea, for example, the December “no touch” training of CHWs came eight months after the first Ebola case was reported in the district.

There was also a great deal of confusion over the intent and practical implementation of the policy. Many interpreted the policy as an instruction to cease treatment entirely and cited this as a reason for stopping services. A number of district- and national-level stakeholders engaged in this study also conceded that the policy had been introduced too late in the response and only after a number of CHWs had died having been infected by Ebola during the course of their work. CHWs also displayed significant variation in their understanding of the implications of the “no touch policy” on their clinical decisions within iCCM. For example, because of the overlapping symptoms of Ebola and iCCM illnesses, there was a lack of clarity for CHWs on when to treat children with fever or diarrhea in the community vs when to refer them to a facility. They felt that the “no touch policy,” and in particular the prohibition on the use of rapid tests for malaria, limited their ability to differentiate children with malaria or diarrhea from suspected Ebola cases. Some CHWs chose to refer all patients to a facility, while others treated all fevers presumptively for malaria and only referred if the patient did not recover after 24-48 hours. Alternatively, some CHWs only referred fever cases if there were other signs or symptoms of Ebola (eg, diarrhea or vomiting). As one CHW in Sierra Leone reported, “*The caregiver was telling us the condition of their child and we could give them drugs based on their explanation, but it was difficult to diagnose the actual sickness the child was suffering from*.”

**Figure 2:**
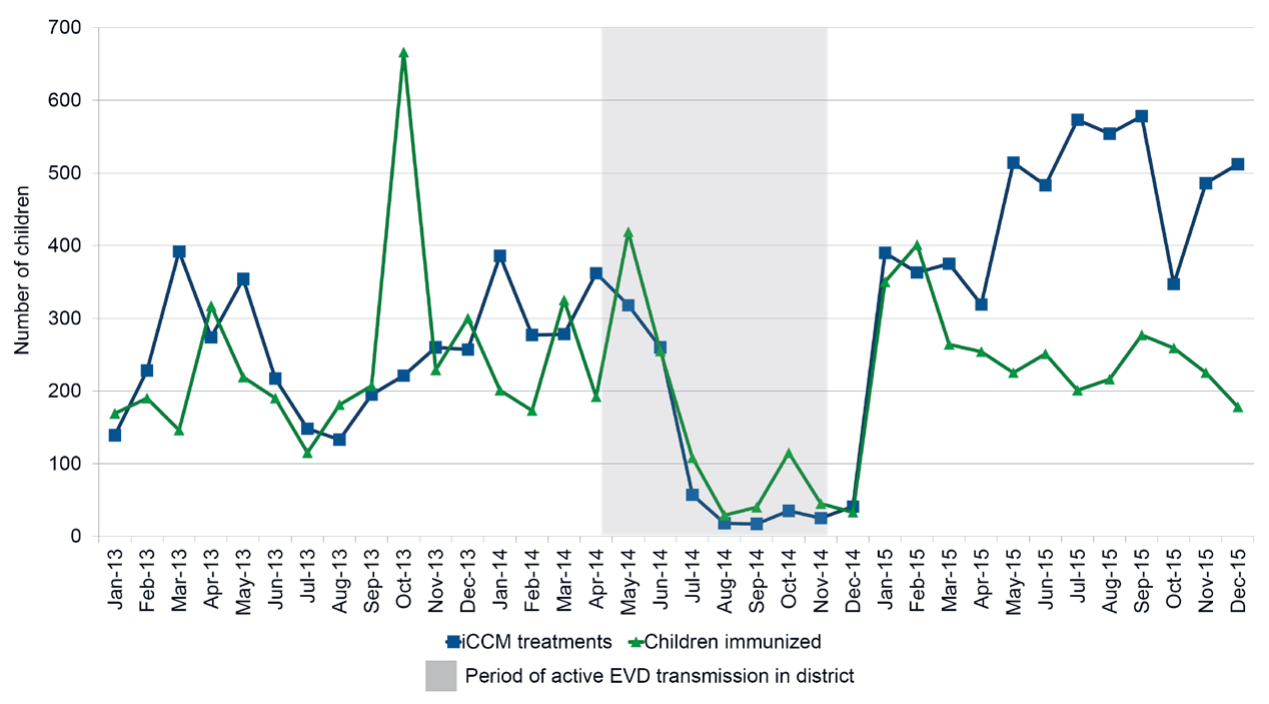
Number of integrated community case managemen (iCCM) treatments and children immunized at community level in Bong, Liberia, January 2013 – December 2015.

As discussed above, iCCM services in three of the study districts in Liberia had largely ceased prior to Ebola. Quantitative data from Bong (**Figure 2**), the one district that had ongoing service provision at the start of the Ebola outbreak, showed that iCCM and immunization services largely ceased during the height of the outbreak in the district, from July to December 2014. By January 2015, there were no more Ebola cases in the district, and the number of treatments recovered to the pre-Ebola level.

**Figure 3:**
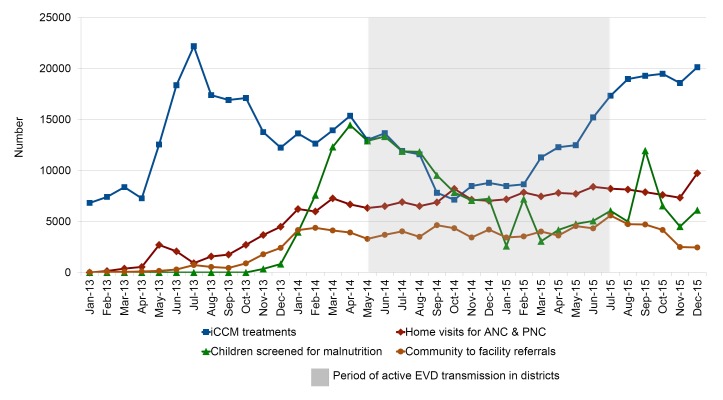
Number of integrated community case managemen (iCCM) treatments, children screened for malnutrition, antenatal/postnatal visits, and referrals at community level in Kenema, Tonkolili, Kailahun, and Bombali Districts (aggregated), Sierra Leone, January 2013 – December 2015.

In Sierra Leone CHWs explained that continuation of routine services was largely dependent on the extent to which their community had been affected by Ebola: in communities with a high Ebola caseload, CHWs tended to focus mostly on EVD-related duties. Quantitative data from the four study districts (**Figure 3**) show that iCCM treatments and malnutrition screening declined, but did not cease entirely, from the early period of the outbreak in June 2014. iCCM services started to recover around December 2014, which was still during the height of the outbreak in the districts, while malnutrition screening continued at lower levels throughout the period observed. In contrast, the number of home visits and referrals to health facilities did not seem to be affected by the outbreak.

In Guinea, CHWs in Macenta, a district that was affected by Ebola earlier in the outbreak, and Kérouané, which bordered Macenta, were instructed to stop all community-based treatment of malaria and refer patients to local health facilities. Dubréka and Forécariah did not record active Ebola transmission until August and September 2014, respectively. By then, implementing partners in both districts had benefited from time to prepare and adjust their activities. Consequently, CHWs were advised to continue CCM using the “no touch” approach and CCM programming largely continued. MNCH promotion activities during Ebola followed a similar pattern; in the districts that were affected earlier in the outbreak, communication and social mobilization activities largely shifted from MNCH to Ebola infection prevention control (IPC). In the districts that were affected later, CHWs reported that they continued MNCH sensitization activities. As one CHW concluded “*Our regular activities on the sensitization of maternal health and child health were not disturbed at all, because any time we go to the field, we start first the sensitization on our malaria, pneumonia, and diarrhea activities, then we add on the Ebola sensitization*.” By early 2015 the intensity of Ebola transmission had eased in Guinea. While the Ebola response was ongoing, partners began re-committing resources to strategies aimed at increasing accessibility and utilization of essential MNCH services. The subsequent expansion of community health services included training CHWs to provide iCCM and screen for malnutrition. From March 2015, CHWs were trained on iCCM with “no touch” modifications across three of the four study districts.

In all three countries, the supply chain and supervision issues that existed prior to Ebola continued or were exacerbated during the outbreak. In areas where CHWs were instructed to continue MNCH services, program implementers faced a number of service delivery challenges in addition to those that existed prior to Ebola. These included NGO policies restricting staff movement for safety reasons; the inability of NGO workers to enter many communities due to hostility and community resistance; the closure of health facilities due to quarantine, illness, or the death of health workers; and an overwhelming preoccupation with Ebola-related activities. CHWs who had not been consistently supervised during the outbreak expressed a keen sense of abandonment. As one CHW in Sierra Leone asserted, “*We went nine months without seeing the partner staff. They used to supervise us and were responsible for paying our incentives. If those things are not provided then how can we work*?”

#### Care seeking and referral compliance

Because of their ties to health facilities, communities displayed elevated levels of fear and mistrust toward CHWs. Many caregivers of ill children avoided CHWs and used traditional remedies or sought care from drug vendors. As one caregiver in Sierra Leone recalled, “*We were afraid of using all medicines from the health facility and the CHWs because we heard that there was an Ebola tablet and if you drink it you will have Ebola. So we were using the traditional herbs instead.”* Caregivers were also afraid of what they perceived to be indiscriminate referral to health facilties or Ebola treatment units (ETUs). As a CHW in Sierra Leone explained,

“The children were suffering from fever and whenever their parents saw us in the community they would hide their sick children. Nobody would complain about being sick to you because they believed that if you were aware of their sick people you would refer them. They were answering us differently...The community people were denying sickness and there was not a good relationship between us.”

It should be emphasized, however, that according to many stakeholders, the long-established close and trusting relationship between communities and their CHWs was significantly more resilient than the relationship communities had with facility-based health workers. Although CHW service utilization declined, caregivers in the three countries confirmed that they were still more likely to seek care from CHWs than from health facilities. Community leaders also emphasized that they were primarily fearful of outsiders, and the provision of effective treatment by CHWs helped to dispel rumors and rebuild community trust in CHWs. As one CHW in Guinea concluded,

“It was not really easy, but we fought a lot because at the beginning of Ebola many people were not coming and they were criticizing us, but when we started treating patients and they were getting well, it was now those patients that were helping us to sensitize the others by their testimonies, telling them that, “You are just afraid for nothing, there is no Ebola with them, we went and got treatment and now we are healthy.””

Stakeholders reported that as communities gradually adopted Ebola IPC measures, the rate of utilization for MNCH services also increased. Stakeholders attributed this to a combination of strategies, including intensive and persistent sensitization by community and youth leaders, religious leaders, health facilities, and CHWs. A CHW in Sierra Leone explained, “*We kept talking to the community that they should please accept the medicine and give it to their children so that they would not die. And they all eventually accepted the message.”*

#### Other community-based actors

As pregnant women started to avoid health facilities, TBAs reported that they conducted increased numbers of home deliveries. However, most TBAs reported that they were given neither training nor IPC materials. The following testimony provided by a TBA in Liberia was broadly illustrative of the role TBAs played in assisting community-based deliveries during the Ebola outbreak.

“We were told not to do what we did during the outbreak, doing the community delivery business, because it was dangerous...I delivered more than ten women during Ebola... We are living in the same community together with the big bellies [pregnant women]. We see them everyday, so we cannot see a pregnant woman crying in labor pain and not go there to help. So I started to use hand gloves if I could find them or to tie plastic on my hands...We needed PPE [personal protective equipment], but we did not have it. It was just by God’s grace that we survived. We needed gloves, rain boots, chlorine, hand sanitizer, soap, buckets. These were things that we needed to work, but we were not given any. Everything was given to the big people and we were left out.”

Authorities perceived that THs could be “super-spreaders” of Ebola, and in response the governments called for a cessation of all traditional healing practices. In Sierra Leone and Guinea, this was bolstered by the implementation of bylaws which prohibited traditional practices. Some THs were trained on Ebola warning signs, encouraged to avoid physical contact with patients, and instructed to refer all ill people directly to health facilities. Nevertheless, many THs highlighted their frustration that they were not incorporated and utilized in the response more quickly, despite often being the first point of contact for patients. According to THs, it was not until “*Ebola was almost finished*” that the governments and Ebola response partners started to train and actively include them in the Ebola response. As one TH in Guinea concluded,

When Ebola started we really saw difficulties because we were not aware of which type of sickness it was.

“*That is what killed many of our friends...It was later on that we had the information that Ebola is a sickness that can kill people. If one person is infected in a family, and if that family is not careful, they will all be infected. So based on that information we started keeping our distance. Along the way, we received a call that all the traditional healers should come for the training. So it was the training that prevented many more of our friends being killed.*”

### Community-level Ebola response

Although CHWs were well established at the community level, they were under-utilized in the initial response to Ebola. Despite this initial lack of formal engagement, CHWs reported that they continued to liaise with health facilities and were a conduit for passing information to their communities, but this was done on an informal and ad hoc basis.

Instead of engaging existing CHWs, many new workers were recruited by district health departments and Ebola response partners to conduct community-level activities. These personnel were usually recruited from outside the communities in which they were deployed, and the selection process was sometimes determined by nepotism, where people connected to decision-makers were selected for relatively well-paying Ebola response roles, even if they were not the most qualified. The deployment of these workers proved problematic and ineffective due to community mistrust and the, at times violent, rejection of outsiders. Over time, it became apparent that new recruits were not well accepted or tolerated by communities if they were not a resident or previously known. In response, CHWs who had been providing MNCH services prior to the outbreak had their duties expanded to include Ebola-related roles, which they conducted either in addition to or in place of their routine MNCH activities.

A primary role that CHWs performed was social mobilization and community engagement on Ebola prevention and control. Activities included raising awareness of Ebola; describing how to avoid Ebola (eg, not to eat bush meat, to avoid contact with an ill person, and to not attend or perform traditional burials); advocating for the adoption of prevention strategies (eg, using chlorine solution and hand washing); and distributing basic materials including buckets, chlorine, soap, hand sanitizer, and gloves. CHWs reported that they went door-to-door, visited farms to engage people while they worked, organized community meetings, and undertook opportunistic sensitization at community gatherings (such as weddings or Friday prayers). Strategies to “demystify” Ebola were also adopted, including the organization of community visits to ETUs to observe patient care, and recruiting survivors to share their experiences and encourage early care seeking.

CHWs also worked as contact tracers, creating lists of all known contacts associated with an Ebola case, searching for the contacts, and isolating and monitoring them to determine if they experienced any signs or symptoms of Ebola. Any contact presenting with signs or symptoms was reported and referred for treatment.

CHWs were also trained and deployed as “active case finders,” carrying out door-to-door searches for symptomatic people in their communities. If they identified somebody who was ill, they would isolate them, report the case to district health officials, and refer them to a health facility or ETU. As one district health official in Sierra Leone explained,

“They were searching for cases, reporting cases and deaths. That was the time when the CHWs were very active because they were still within their communities. Where they stay, they can know about people who are sick and hidden. They even report to us about people who died secretly. The CHWs...were very useful at that point in time.”

Many CHWs also reported becoming “caretakers,” assisting suspected Ebola patients while waiting for the ambulance to collect and transfer the patient to a health facility or ETU. CHWs were called on to perform this role when other community members were too afraid to come near the patient. Although some CHWs reported receiving training on how to care for somebody with EVD in the community, most had not received specific guidance.

As mentioned above, CHWs’ association with health facilties, the government, and international organizations, and their engagement as Ebola responders, resulted in significant fear, mistrust, and rejection by communities. Across the study districts, CHWs reported experiencing stigmatization as potential “Ebola carriers”; accusations of witchcraft; accusations that they were spreading the virus; harassment from families of symptomatic patients they had reported to authorities; mistrust from community members who hid their health status or refused care; anger from their own family members that their work put them and their household at heightened risk; and anger from their family and community for not fulfilling responsibilities such as farming and harvesting due to Ebola-related work. As one CHW in Guinea stated, “*During Ebola it was difficult to provide our services because many people didn’t believe or trust us, the community members were insulting us, abusing our mothers, throwing stones at us, and considering us to be Ebola carriers.”* Another CHW in Liberia recalled, “*It is true that people were afraid of us. Even when we were walking in the community and they saw us they used to insult us and tell us that we were the people that were eating the government money so that we could kill their people in the community...When you ask them to go to the hospital they will not agree to do so.*” Many CHWs recalled feeling discouraged when they thought they had successfully conveyed key prevention and protection messages, yet no behavior change resulted.

Mistrust in the health system, compounded by initial messaging that Ebola was fatal and untreatable, contributed to the slow uptake of the Ebola messages delivered by CHWs. People who displayed symptoms frequently attempted to evade detection during the outbreak, often hiding on their farms or in the bush, and were resistant to being referred to a health facility or ETU. Some believed rumors that health facility staff deliberately infected patients with Ebola. As one caregiver in Sierra Leone recalled,

“*We were afraid of using all medicines from the health facility and the CHWs because we heard that there was an Ebola tablet and if you drink it you will have Ebola*.”

Other caregivers recalled how their loved ones were “*carried away blindly”* to distant treatment centers. The fear of ambulances led to violence in some communities. In Dubréka Prefecture in Guinea, ambulances were set on fire by angry and frightened community members. As one community leader in Guinea asked,

“If you hear that a car kills someone, or you saw a car kill somebody, when you see a car coming another time will you stand or will you run away?”

Community members also stated they were reluctant to adopt the Ebola-related practices that conflicted with cultural norms. A caregiver in Guinea explained,

*“It was very hard for us to accept the messages they were giving us to isolate our patients. Because according to our tradition, when a family member is sick, we will come close to him, cherish him, feed him, wash him*.”

Despite these challenges, the pre-existing relationships between CHWs and communities helped in re-establishing trust during the emergency period, and CHWs were more effectively able to carry out highly sensitive Ebola-related activities than outsiders. As one community leader in Guinea explained,

“*Our CHW was determined even during Ebola. He stood up and said he was born here and grew up among us and so we should not fear him because he would stand for us, and would not harm us in any way, and instead we should work together to chase out this sickness of Ebola.”*

A national-level stakeholder in Guinea further explained,

“Many community workers that were chosen outside the communities were beaten and chased away, but when we selected CHWs from within the communities, they were accepted and our messages were accepted since these CHWs were trusted family members. Through the CHWs we were able to locate and get hold of suspected Ebola patients who had run away out of fear because they are part of them as family members, making it easier for the CHWs to know where they had hidden themselves.”

Many stakeholders, including community members, recognized that CHW activities played a major role in building trust between communities and the health system and in breaking chains of transmission. As an Ebola survivor in Sierra Leone concluded, “*If it hadn’t been for the efforts of the CHWs, all of the people would have got Ebola in this community because the CHWs were going house-to-house, telling* [ill] *people to go to a health facility*.”

#### Other community-based actors

At the community level, the mobilization of “Ebola task forces” or “community watch committees” was seen as a useful mechanism to quickly coordinate response efforts and enforce protocols at the community level. Members of the task forces included community, religious, womens, and youth leaders; THs; CHC representatives; and CHWs. Members were specifically chosen by the community to ensure that those most resistant to Ebola response activities were included. As one district health representative in Guinea explained, *“We attacked the problem at the source by training and including...youths, since youths were throwing stones at Ebola workers. And we included women leaders since women were often against safe burials conducted by the Red Cross.”* Amongst other actions, the task forces oversaw community-based surveillance (for example monitoring for strangers or symptomatic people entering the village), active case finding and referral, promoting safe and dignified burials, establishing hand washing stations, promoting compliance with the isolation and referral of ill people, and taking care of orphans. All of these activities included social mobilization on the prevention of Ebola, and dispelling or debunking “Ebola rumors.”

CHCs were a natural platform for the establishment of Ebola task forces and subsequent coordination activities, and stakeholders suggested that communities with a CHC in place prior to the Ebola outbreak appeared to be more resilient and were more likely to self-mobilize and organize community-led solutions. CHC members described their roles during the response as similar to those of CHWs. The main difference was that CHC members undertook these activities largely without formal support. As one CHC member in Liberia emphasized,

“When all of the others left, we, the community structures, had to stay to work within our community to make sure that this virus did not finish everybody. We [the CHC] met the county health team and told them that the people they sent into the community were afraid of going to affected areas, so we asked them to train us instead of training other people from outside.”

Despite the trust that communities felt for TBAs, they were rarely included in the formal EVD response because they were considered not sufficiently skilled to take on Ebola-related activities. Similarly, THs were sidelined throughout much of the formal response. In some hotspot areas, THs were eventually trained and supported to work as social mobilizers, but this collaboration came late in the outbreak.

### Community-based MNCH policies and services post-Ebola

#### Service delivery

As the countries transitioned into the post-Ebola recovery period, Ebola-related activities were scaled back and implementing partners focused again on MNCH programming. Partners worked quickly to strengthen community-based MNCH services where they had continued throughout the outbreak and to restart services where they had ceased. While some organizations had returned to “business as usual,” with implementing partners continuing to run short-term programs, often in parallel to the public health system, other partners were scaling up more comprehensive community health packages in line with new national policies.

Implementing partners discussed a number of ongoing challenges in implementing community-based MNCH services after the outbreak. Drug stock-outs remained a key challenge to service provision, as before Ebola. Stakeholders in all three countries confirmed that when CHWs were re-activated and when new iCCM services were established post-Ebola, CHWs were given initial stocks of drugs for the first month or so. However, once the initial stocks were depleted, CHWs experienced frequent and persistent stock-outs. CHWs in most of the study areas reported that widespread drug stock-outs prevented them from providing community-based treatment at the time of the study.

Stakeholders also confirmed that challenges in supervision and monitoring of CHWs were similar in the post-Ebola period to those experienced before the outbreak, particularly because some of the management structures that were put in place during the response had since been removed. Program implementers highlighted that the lack of dedicated personnel to supervise CHWs was detrimental to the successful implementation of iCCM.

#### Care seeking and referral compliance

With the restart of routine services, the MoH and partners implemented social mobilization and community engagement strategies to rebuild trust, increase utilization of services, and improve referral compliance. Respondents suggested that the mistrust communities had harbored for CHWs during the Ebola outbreak had largely resolved. In a number of villages, participants reported a greater depth of respect and trust for their CHWs because of the efforts made by CHWs to protect them during the Ebola outbreak. Many CHWs asserted that care seeking for childhood illness had almost returned to pre-Ebola levels in areas where CHWs had iCCM drugs available. A CHW in Sierra Leone concluded,

“*We are now working amicably with the community and they have realized that they were just blaming us for nothing during the outbreak. They are no longer hiding sick children from us and they will knock at our doors at any time if they have a sick child.”*

Officers-in-charge at health facilities also reported that patient numbers had returned to normal and most caregivers confirmed that they would attempt to comply with referrals made by their CHW.

However, the drug supply issues described above severely impacted both CHW motivation levels and community perceptions of CHW services. Many caregivers cited drug stock-outs as a determining factor in their decision to seek care from THs or other informal providers. Community-level stakeholders emphasized that the lack of medicines held by both CHWs and health facilities persistently caused delays in treating ill children. As one community leader in Guinea concluded,

“One of the greatest problems we face here is the lack of medication. Sometimes we go to the traditional healer and the treatment doesn’t work. Since the CHW has no drugs, we go to the health post. The health worker may be willing to offer the treatment, but the drugs aren’t available. So the patient lies waiting while the health worker takes his mother’s bike to get medication for the treatment. The waiting causes more harm to the patient because before the health worker returns the patient becomes more ill.”

In Guinea, the continuation of a cost-recovery policy meant that community members were required to purchase antibiotics, zinc, and oral rehydration salts from CHWs. While CHWs appreciated the financial benefit, they were concerned that this strategy was affecting caregiver perceptions of CHWs and utilization of their services. In some areas, caregivers reportedly turned to purchasing drugs at lower cost in the market. Some CHWs also reported that caregivers would “boycott” CHWs in protest of having to pay for drugs. Highlighting the distrust this policy caused, one CHW in Guinea explained,

“We are really suffering with the pneumonia and diarrhea drugs because they are to be sold in the community to the patients. But many of them are refusing to buy them from us, they prefer going to the pharmacy to buy it [even if it’s more] expensive. They think that we are just selling these drugs for our own interest, that these drugs are not meant to be sold to them, that they should be free of charge like malaria drugs. So many of them are not coming to us for those treatments.”

#### CHW remuneration and motivation

The issue of remuneration for CHWs was one of the most hotly debated topics among stakeholders at all levels in this study. In recognition of concerns about the sustainability of community health services, all three countries had developed new community health policies and strategies that call for national cadres of well-trained, paid CHWs providing a broad package of services. The policies in Sierra Leone and Liberia call for monthly payments of around US$ 25 and US$ 70, respectively, for salary and transportation costs. In Guinea, the policy did not specify an amount, but a key MoH representative confirmed that the government intended to implement a policy requiring all CHWs be paid a monthly payment of US$ 60. Stakeholders at all levels were in strong support of the proposal to pay CHWs, but they also expressed concerns over the feasibility of implementing this policy. The current model of external donor funding was not seen as a viable or sustainable option for supporting a large-scale, institutionalized community health program. A number of national stakeholders suggested that CHW salaries would have to be funded primarily through the government payroll, which would also strengthen accountability and oversight. However, some participants also questioned the viability of paying CHWs through the government payrolls, which did not yet have the capacity to remunerate facility workers regularly, and were plagued by corruption and a lack of transparency.

For their part, CHWs clearly articulated their dissatisfaction with having to return to volunteerism, especially after the experience of receiving higher payments for Ebola-related activities. Many CHWs suggested that they would consider leaving the position if they were not better incentivized in the future. As one CHW in Guinea concluded, “*A time will come when we have to first take care of our families and make sure they are fed and survive, so we will one day see someone dying and we will not attend to the person because we will be attending to our families*.”

They also resented that the government had afforded them little recognition for their contribution to the Ebola response. As one CHW in Sierra Leone explained,

“They gave us confidence that they would give us a package at the end of the Ebola fight. For example, ...they [some CHWs] would be given a scholarship or the government would enroll those who wanted to work in the Republic of Sierra Leone Armed Forces or Sierra Leone Police. But at the end of Ebola, they never said thank you to us, and they isolated us after we had fought the Ebola war.”

Less controversial were discussions regarding non-financial incentives. These included preferential selection for paid work to implement health campaigns, opportunities for scholarships to facilitate career development, financing education for CHWs’ children, and micro-financing for small businesses. CHWs regarded all these initiatives to be positive and desirable.

#### Other community-based actors

Across the three countries, many stakeholders, particularly community leaders and CHWs, emphasized the need to formally recognize TBAs and CHC members and their important role in the provision of community-level health services in the post-Ebola period. As part of the recovery process, there was a renewed focus on establishing or re-activating CHCs to ensure that communities have a role in the planning, implementation, financing, and monitoring of community health initiatives.

Many TBAs who had performed an increased number of home deliveries during the Ebola outbreak had ceased conducting deliveries. However, home deliveries were still taking place, particularly in the hardest-to-reach communities. TBAs emphasized that after the outbreak was declared over, they had not received any formal acknowledgement of their role during the response. As one TBA in Liberia concluded, “*We were not even called to say thank you.*” They were keen to continue working, particularly with CHWs, but stressed the need for recognition and remuneration.

THs also resented their marginalization during the Ebola response, the ongoing decrees prohibiting use of THs, and persistent efforts of CHWs to discourage the utilization of THs. They expressed a firm desire to be integrated into the health system and maintained that they complemented the efforts of health workers and CHWs by offering treatment for conditions not suited to biomedical interventions, including psychological illness. District-level stakeholders also recognized that community members were likely to continue seeking care from THs for a range of illnesses, and acknowledged the need for them to be included in community health plans. They suggested, for example, that THs had a role to play in community-based surveillance or community sensitization.

## DISCUSSION

The results of this study demonstrated mixed results in terms of resilience of community-based MNCH services. There was clearly a sharp decline in service provision in the early months of the Ebola outbreak due to weak service delivery, confusion over policy, and the overwhelming nature of the Ebola outbreak. However, the majority of CHWs remained active in their communities and were willing to continue providing services, and many did so in the early days of the outbreak without formal direction or remuneration. When CHWs received clear directives to restart case management services, were trained on the “no touch policy,” and were provided with drug supplies, service provision rebounded, even during the outbreak. Non-clinical services, which did not carry the same risk as clinical care and did not require a continued supply of drugs, appear to have been less affected.

Although the implementation of the “no touch policy” allowed iCCM services to restart, there were a number of complications with this policy that should be addressed by global and national policy makers. First, the policy must be more clearly communicated to implementing partners and CHWs. Second, the training of CHWs on the policy must do a better job of clearly communicating the treatment protocol. Third, there is a need for assessments of the quality of care provided by CHWs using the “no touch policy” to assess whether CHWs are following the guidelines, to evaluate the balance of risk vs benefit of implementing the policy, and to assess the effect of the policy on utilization of services.

These results are consistent with other studies [16-21] in showing that the late engagement of CHWs and other community actors hindered the outbreak response. Outsiders sent to affected communities were rejected by communities and some efforts to impose changes on communities were counter-productive. Although CHWs faced mistrust and stigma because of their ties to health facilities, they were eventually able to gain the trust of community members because of their longstanding relationships.

In addition to CHWs, this study showed the importance of engaging other key community actors. Engagement of trusted and respected community leaders was crucial to mounting an effective community-level response, and the existence of active CHCs facilitated community mobilization and coordination. In contrast, TBAs and THs gained increased prominence as trust in health workers diminished, but they were not adequately engaged in the response. In an emergency, all of these community actors should be immediately engaged in a coordinated response.

Following the Ebola outbreak, stakeholders at all levels underscored the importance of strong community-based health systems to achieve increased and more equitable coverage of essential MNCH interventions, and to improve resilience of health systems. The national community health policies in all three countries provide strong foundations for strengthening the community health systems. However, it is still unclear how these policies will be operationalized and financed. Furthermore, there remain critical service delivery weaknesses, particularly regarding supply chain, supervision, and transportation for referral, that were present before, during, and after Ebola. It will require a long-term commitment to health system strengthening at all levels to address these challenges.

### Implications for building resilience for future emergencies

A number of lessons can be drawn from these findings.

First, affected communities should be seen not just as victims, but as essential partners in both preparedness and emergency response. Response strategies should be developed with their input and delivered through trusted community actors and structures.

Second, the structure for community-based response should be established prior to an emergency. The presence of trained CHWs, TBAs, and CHCs prior to Ebola all contributed to a more effective response in their communities. During the outbreak, the response suffered because linkages between the health system and community health actors were not well delineated before the outbreak.

Third, guidance to CHWs and other community actors needs to be clear and consistent. This will be facilitated by the development of emergency preparedness and response plans detailing the roles and responsibilities of different actors and lines of reporting through the health system.

Fourth, until quality services are consistently delivered during non-emergency times, it will be unrealistic to expect better results during emergencies. Furthermore, it will be impossible to build trust in the health system without making quality services consistently available.

Fifth, service provision and quality of care will only be improved if they are measured. Rigorous and routine assessment of the strength of program implementation (eg, training, supervision, drug supplies, accessibility) and quality of care should be included in countries’ monitoring and evaluation plans moving forward.

Sixth, solutions to the challenge of financing the scale-up and institutionalization of community health services in an era of declining international resources must be developed. This will likely entail a combination of increased government resources committed to health and a reprioritization of international assistance toward long-term support for institutionalizing community health services as part of a strong national health system [22].

Seventh, with acknowledgement of the difficulties of carrying out research in emergencies, there is an urgent need for rigorous implementation research and evaluation of health interventions in emergencies [23]. A real-time evaluation of the effectiveness of the work of CHWs during the Ebola outbreak would have provided invaluable information to improve services in future emergencies.

### Limitations

There were several limitations to this study. First, in qualitative research, there is a risk of misinterpretation and the possibility that respondents provide what they perceive to be socially desirable answers or withhold sensitive information. Second, due to poor road conditions, it was not possible to access remote villages located more than 2.5 hours from the central town. This may have positively biased responses relating to health seeking behavior and referral compliance. Third, CHW reporting rates for MNCH activities reportedly declined during the outbreak, which may have led to under-reporting of activities. Finally, quantitative program data were checked for completeness and consistency, but it was not possible to verify the accuracy of data. Furthermore, quantitative data were used for only five of the 12 study districts because data from other districts were either not available or did not pass the completeness and consistency check.

## CONCLUSIONS

CHWs, TBAs, CHCs, community leaders, and THs all played important roles during the Ebola outbreak. However, the ability to maintain routine services and the effectiveness of the Ebola response were hampered by the fact that these community-based health actors were engaged late in the response and did not receive sufficient support. In the future, CHWs and other community-level actors should be placed at the forefront of emergency preparedness and response plans and they must be adequately supported to strengthen service delivery.
